# The Influence of Dopaminergic Striatal Innervation on Upper Limb Locomotor Synergies

**DOI:** 10.1371/journal.pone.0051464

**Published:** 2012-12-07

**Authors:** Ioannis U. Isaias, Jens Volkmann, Alberto Marzegan, Giorgio Marotta, Paolo Cavallari, Gianni Pezzoli

**Affiliations:** 1 LAMB P.&L. Mariani, Section of Human Physiology Department, Università degli Studi di Milano, Milano, Italy; 2 Centro per la Malattia di Parkinson e i Disturbi del Movimento, CTO, ICP, Milano, Italy; 3 Neurologische Klinik und Poliklinik, Universitätsklinik Würzburg, Würzburg, Germany; 4 Dipartimento di Medicina Nucleare, Fondazione IRCCS Ca' Granda Ospedale Maggiore Policlinico, Milano, Italy; INSERM/CNRS, France

## Abstract

To determine the role of striatal dopaminergic innervation on upper limb synergies during walking, we measured arm kinematics in 13 subjects with Parkinson disease. Patients were recruited according to several inclusion criteria to represent the best possible *in vivo* model of dopaminergic denervation. Of relevance, we included only subjects with normal spatio-temporal parameters of the stride and gait speed to avoid an impairment of upper limbs locomotor synergies as a consequence of gait impairment *per se.* Dopaminergic innervation of the striatum was measured by FP-CIT and SPECT. All patients showed a reduction of gait-associated arms movement. No linear correlation was found between arm ROM reduction and contralateral dopaminergic putaminal innervation loss. Still, a partition analysis revealed a 80% chance of reduced arm ROM when putaminal dopamine content loss was >47%. A significant correlation was described between the asymmetry indices of the swinging of the two arms and dopaminergic striatal innervation. When arm ROM was reduced, we found a positive correlation between upper-lower limb phase shift modulation (at different gait velocities) and striatal dopaminergic innervation. These findings are preliminary evidence that dopaminergic striatal tone plays a modulatory role in upper-limb locomotor synergies and upper-lower limb coupling while walking at different velocities.

## Introduction

Upper limb locomotor synergies are a basic component of human gait. They represent an active phenomenon driven by locomotor centers within spinal cord and the brainstem and modulated by cortical and subcortical inputs [1], [2]. Information on neural control of locomotor automatism and gait-related arm motion in humans is still scanty. Animal studies, as well as preliminary functional imaging studies in humans, indicate that locomotor movements are coordinated by spinal networks referred to as a central pattern generators (CPGs), which are governed by the brainstem locomotor command region that, in turn, is under the control of the basal ganglia and premotor cortex [3]–[9].

Parkinson disease (PD) is a progressive neurological condition characterized by bradykinesia, rigidity, postural instability and possibly tremor. Patients with PD typically show little or no arm oscillation while walking, and this is often the first clinical motor sign to appear [10], [11]. In PD patients, the arm swing is not correlated with clinically tested shoulder rigidity [10] and often disproportional to the degree of upper limb akinesia during voluntary alternating movements, thus pointing to a task-specific motor disturbance associated with walking. At an anatomopathological level, PD is mainly characterized by the loss of dopaminergic neurons in the substantia nigra pars compacta, which leads to striatal dopamine depletion [12]. It has been estimated that motor PD symptoms appear when the loss of dopamine neurons reaches the 50% to 60% threshold, [13] which corresponds to a 70% to 80% decrease in putaminal dopamine content [14].

The aim of this study is to investigate a putative role of dopamine and the striatum in locomotor upper limb automatism, taking into account their relationship with walking speed. Patients were carefully selected to represent a clinically homogenous *in vivo* model of dopaminergic striatal innervation loss (see, *Subjects*) which was measured by [^123^I] N-ω-fluoropropyl-2β-carbomethoxy-3β-(4-iodophenyl) tropane (FP-CIT) and single-photon computed tomography (SPECT).

## Materials and Methods

### Ethics Statement

The local institutional review board (Section of Human Physiology, DePT) approved the study and all patients provided written informed consent.

### Subjects

We tested 13 subjects with idiopathic PD (six males; mean age: 64 years; range: 52–73 years; disease duration mean: 5 years; range: 3–6) and a control group (HC) of 10 neurologically healthy adults (seven males; mean age: 64 years; age range: 55–70 years). The diagnosis of PD was made according to the UK Parkinson Disease Brain Bank criteria and patients were clinically evaluated by means of the Unified Parkinson Disease Rating Scale motor part (UPDRS-III; range: 0–108). Median UPDRS-III score was 21 (range: 11–32). A sub-score for unilateral arm rigidity and bradykinesia (UPDRSrb) was calculated as the sum of UPDRS items 22 (rigidity), 23 (finger taps), 24 (hand movements), 25 (rapid alternating movements of hands). Median UPDRSrb score of the worst arm was 8 (range: 4–11) and of the less affected arm was 4 (range: 1–5). We selected only mildly affected patients for this study (Hoehn & Yahr II) (see later).

At the time of the study, and during a follow-up time of at least six months after enrollment, no patient showed any signs indicative for atypical parkinsonism (e.g. gaze abnormalities, autonomic dysfunction, psychiatric disturbances, etc.). All patients reported a marked improvement (>30% UPDRS-III score reduction) after the intake of L-Dopa or dopamine agonists. L-Dopa daily dose and L-Dopa Equivalent Daily Doses (LEDDs) were also recorded, with the latter calculated according to the following conversion ratio: 100 mg levodopa = 1.5 mg pramipexole = 6 mg ropinirole. Median value of LEDDs per day was 500 mg (range 200–625 mg).

All subjects were screened for cognitive impairment by the Mini-Mental State examination, Clock Drawing Test and Frontal Assessment Battery and excluded if not meeting normal, age-related performance.

Other exclusion criteria for study participation were a history of neurological disorders (other than PD for patients), head trauma with loss of consciousness, orthopedic diseases, systemic illness or previous orthopedic, brain or spinal cord surgery. A MRI was performed within six months from enrollment and only subjects with normal results (i.e., no sign of white matter lesion or atrophy) participated in the study.

Gait disturbance is a key component of motor disability of PD and patients may variably present with reduced gait speed, shortened stride length, prolonged stance and double support phases [15]. At an early disease stage, PD patients may still show normal spatio-temporal gait parameters during steady linear walking [16]. To avoid an impairment of upper limbs locomotor synergies as a consequence of gait impairment *per se,* we enrolled only PD patients with normal spatio-temporal parameters of the stride (Table 1).

**Table 1:** Spatio-temporal parameters of the stride, arm ROM and phase shift of subjects with Parkinson disease and healthy subjects.

	Parkinson patients		Healthy subjects	
Gait speed (Km/h)	3.81 (1.86, 7.81) (preferred gait speed range: 2.87, 4.35)		3.82 (1.24, 9.94) (preferred gait speed range: 3.35, 4.4)	
	Contralateral	Ipsilateral	Contralateral (Right)	Ipsilateral (Left)
Arm ROM (°)	7.48 (1.47, 32.06)**	18.19 (2.11, 29.8)**	25.27 (2.29, 31.8)	25.71 (5.21, 38,15)
Phase shift (%stride)	−15.35 (−23.1, −5.6)*	−11.8 (−16.9, −6.1)	−10.17 (−19.1, −5.4)	−9.97 (−18.9, −3.5)
Stride length (mm/BH)	0.68 (0.55, 0.28)	0.68 (0.49, 0.82)	0.7 (0.57, 0.82)	0.69 (0.54, 0.81)
Stride time (sec)	0.89 (0.73, 1.47)	0.92 (0.75, 1.42)	0.86 (0.8, 1,28)	0.87 (0.64, 1.28)
Stance (%stride)	65.2 (58.78, 71.25)	66.56 (62.77, 72.84)	64.42 (61.76, 72.6)	64.28 (61.54, 81.82)
Slope arm ROM[°/(km h^−1^)]	2.08 (0.12, 6.68)	3.47 (0.43, 7.82)	3.96 (2.11−6.34)	3.5 (1.24−6.58)
Slope phase shift[%stride/(km h^−1^)]	4.98 (2.9, 11.6)*	3.85 (2.52, 9.6)*	3.04 (1.55−4.68)	2.88 (1.8−4.16)
Slope stride length[mm/BH/(km h^−1^)]	0.07 (0.05, 0.09)	0.08 (0.05, 0.09)	0.06 (0.05−0.08)	0.07 (0.05−0.08)
Slope stride time[sec/(km h^−1^)]	−0.18 (−0.3, −0.12)	−0.17(−0.3, −0.11)	−0.16 (−0.22, −0.09)	−0.16 (−0.23, −0.09)
Slope stance[%stride/(km h^−1^)]	−0.18 (−0.29, −0.12)	−2.0 (−3.7, −1.4)	−0.15 (−0.22, −0.1)	−1.86 (−2.55, −1.23)

Median values, non-outlier min-max, and levels of statistical difference (Mann-Whitney U-test or Matched pairs) are reported. Data refer at walking at different velocities unless otherwise specified.

* p<0.05 (PD vs. HC); ** p<0.01 (PD vs. HC). BH = body height (mm). ROM = range of motion; Phase shift = temporal delay (%stride) between the positive peak (antero-posterior swing) of the wrist and the negative peak of malleolus; Stride = the period from initial contact of one foot and following initial contact of the same foot, is one gait cycle. Stance = gait phase when a foot is in contact with the ground, it begins with initial heel contact and ends with toe off.

For Parkinson patients, ipsilateral and contralateral refers to the more dopamine depleted hemisphere. For healthy controls, left hemibody refers conventionally to ipsilateral.

### Experimental Protocol and Data Processing

After a 3-day washout of antiparkinsonian medication, subjects performed three sets of six walking trials, at a self-chosen “preferred” speed, “slow” and “fast” speed, in random order along a 10 m path, following verbal instruction in the absence of external feedback. Set-up and data processing has been extensively described elsewhere [16], [17]. In brief, kinematics of body segments were measured during walking, using an optoelectronic system (SMART, BTS, Milan, Italy, sampling frequency 60 frames/s), which computed the 3D coordinates of spherical markers (15 mm diameter) attached on bony fixed landmarks. The marker coordinates were low-pass filtered (cut-off frequency 3–7 Hz, self-estimated by a linear-phase autoregressive model-based derivative assessment algorithm). Specific sets of parameters for the characterization of each task were automatically extracted by *ad hoc* algorithms and then visually inspected to check for possible errors. In particular, the time course of the angular displacement of the humerus segment of the arm with respect to the vertical (positive forward) [17] were computed in planes perpendicular to the inter-acromion line. These measures allowed to analyze the pendular behavior of the arm segment independently from shoulder and pelvic girdle horizontal rotation associated with trunk torsion. Angular profiles were normalized in time as a percentage of the stride duration, and for each cycle we extracted the range of motion (ROM) of absolute arm angle. Finally, arm swing asymmetry (ASA) was calculated according to Zifchock and coll. [18] as follows: ASA = (45°-arctan(ArmSwing_LEFT_/ArmSwing_RIGHT_))*100/90°.

For arm ROM larger than 3°, the phase-shift (upper-lower limb) was further computed as the temporal delay between the positive peak (antero-posterior swing) of the wrist and the negative peak of malleolus, between 20% and 80% of the stride cycle. When the upper limb produced two oscillations per stride, which may occur at the lower walking speeds, phase-shifts were computed using the first positive peak of arm oscillation.

For each subject all variables (e.g. gait speed, arm ROM, phase-shift, etc.) were averaged over trials.

To characterize the speed-related effects, the slopes of the regression lines of arm ROM, phase-shift as well as of spatio-temporal parameters of the stride were computed for each subject as a function of the gait speed.

### SPECT Data Acquisition, Processing and Analysis

Dopamine-transporter (DAT) values were measured by means of Single Photon Computed Tomography (SPECT) with [^123^I] N-ω-fluoropropyl-2β-carbomethoxy-3β-(4-iodophenyl) tropane (FP-CIT).

SPECT data acquisition and reconstruction has been described in details elsewhere [19]. In brief, intravenous administration of 110–140 MBq of FP-CIT (DaTSCAN, GE-Healthcare, UK) was performed 30–40 minutes after thyroid blockade (10–15 mg of Lugol oral solution) in PD patients subsequently overnight withdrawal of dopaminergic therapy. Data were compared with a group of 15 healthy subjects (four males; mean age, 62; age range: 44–70 years).

Brain SPECT was performed by means of a dedicated triple detector gamma-camera (Prism 3000, Philips, Eindhoven, the Netherlands) equipped with low-energy ultra-high resolution fan beam collimators (4 subsets of acquisitions, matrix size 128×128, radius of rotation 12.9–13.9 cm, continuous rotation, angular sampling: 3 degree, duration: 28 minutes).

Brain sections were reconstructed with an iterative algorithm (OSEM, 4 iterations and 15 subsets) and then processed by 3D filtering (Butterworth, order 5, cut-off 0.31 pixel-1) and attenuation correction (Chang method, factor 0.12).

FP-CIT uptake values for the caudate nucleus and putamen of both PD patients and healthy subjects were calculated according to Basal Ganglia Matching Tool [20].

Striatal uptake values were used to calculate an asymmetry index (AI), as follows: AI = (VOI_LEFT_ – VOI_RIGHT_)/(VOI_LEFT_+VOI_RIGHT_)*200.

### General Statistical Analysis

Distribution was non-normal for most of the variables, as assessed by Shapiro-Wilk’s test. Accordingly, descriptive statistics and comparisons were always based on median/range values and non-parametric tests, respectively.

ChiSquare was used to test demographic homogeneity among groups regarding gender.

To relate comparisons to DAT binding values, upper and lower limbs were also re-classified into ipsilateral and contralateral according to the putamen with greater dopaminergic innervation loss. For healthy controls, left hemibody refers conventionally to ipsilateral.

Differences in spatio-temporal gait parameters, arm ROM, phase shifts indexes and slope parameters between control and patient groups were analyzed by means of Wilcoxon two-sample test.

To quantify the (in)consistency of these measures and the stride-to-stride variability, we calculated the coefficient of variation (CV) of these measures when walking at preferred gait speed.

When comparing two hemibodies of the same subject (both for kinematic as well as DAT binding values) we applied a Wilcoxon matched pair test.

Hoeffding's D measure was used to identify correlations among DAT binding values and biomechanic data (including asymmetry indexes) that differed among patients and healthy controls. If no linear correlation was found, we applied partition analyses in search for a DAT binding cut-off value related to abnormal kinematic parameters.

Statistical analyses were performed with the JMP statistical package, version 8.0.2 (SAS Institute, Inc., Cary, NC, USA).

## Results

No difference was found among PD patients and HC for gender distribution and age.

### Imaging Findings

In comparison to HC, patients showed reduced DAT binding values in the putamen (PD, right median: 2.19; right range: 1.2–3.51; left median: 2.63, left range: 1.41–3.4; HC, right median: 4.94, right range: 3.07–5.71; left median: 4.94, left range: 2.96–5.71; p<0.01 all) and caudate nucleus (PD, right median: 4.06; right range: 2.74–5.49; left median: 4.17, left range: 2.3–4.94; HC, right median 5.16, right range: 3.18–6.48; left median: 5.05, left range: 3.07–6.7; p<0.05 all) thus further supporting the clinical diagnosis of PD. In HC, no difference was described when comparing DAT binding values of right and left hemisphere. In PD patients, DAT binding values of the most affected putamen (median: 1.97; range: 1.2–3.07) were on average 30% lower than in the opposite hemisphere (median: 2.85; range: 1.86–3.51; p<0.01). No statistical difference was instead found when comparing DAT binding values of the caudate nucleus of PD patients (most affected, median: 3.73; range: 2.3–4.83; less affected, median: 4.2; range: 2.74–5.5). Average AI value for the putamen of PD patients was 30 (range 6–59); all HC had a putamen AI score below 5 (putamen AI score average 2.1; range 0–4).

UPDRSrb score was negatively correlated with striatal DAT binding values (p = 0.01, RSquare = 0.20), but this relationship explained only 20% of the variance. This finding is in agreement with previous results of SPECT and FP-CIT in subjects with PD and confirms the validity of the methods applied in this study [21].

### Walking at Preferred Speed

As expected, no difference was found for lower limbs spatio-temporal gait parameters (i.e. stride length, stride time and stance) between patients and controls at preferred gait speed (see, Subject).

In HC, right and left hemibodies did not show any difference for any gait-related parameters (Table 1).

Consistency of the spatio-temporal measures did not differ significantly between the patient and control group.

When arms were re-classified into ipsilateral and contralateral to the more dopamine depleted hemisphere (see, General statistical analysis), the contralateral arm ROM of PD patients was reduced when compared to the ipsilateral one (trend towards significance, p = 0.07; Table 1). Contralateral arms also showed a significant anticipation of maximum arm flexion (forward movement) in relation to ipsilateral thigh extension (backward movement), compared to controls (Table 1).

Of relevance, only four subjects with PD showed reduced arm ROM bilaterally. The remaining nine patients had one arm with ROM in the range of normality (ROM>18° [mean ROM_HC_-1SD ROM_HC_]). In the latter group of patients, all but two arms with reduced ROM were contralateral to the putamen with lower DAT binding values. Still, the putamen corresponding to the arm with ROM in the range of normality had over 45% DAT binding loss (with respect to the median value of our normal subjects).

No linear correlation was found between arm ROM reduction and dopaminergic innervation loss. A partition analysis revealed a 80% chance of reduced arm ROM when putaminal DAT binding value was below 2.63 (>47% reduction with respect to median value of normal subjects).

**Figure 1:**
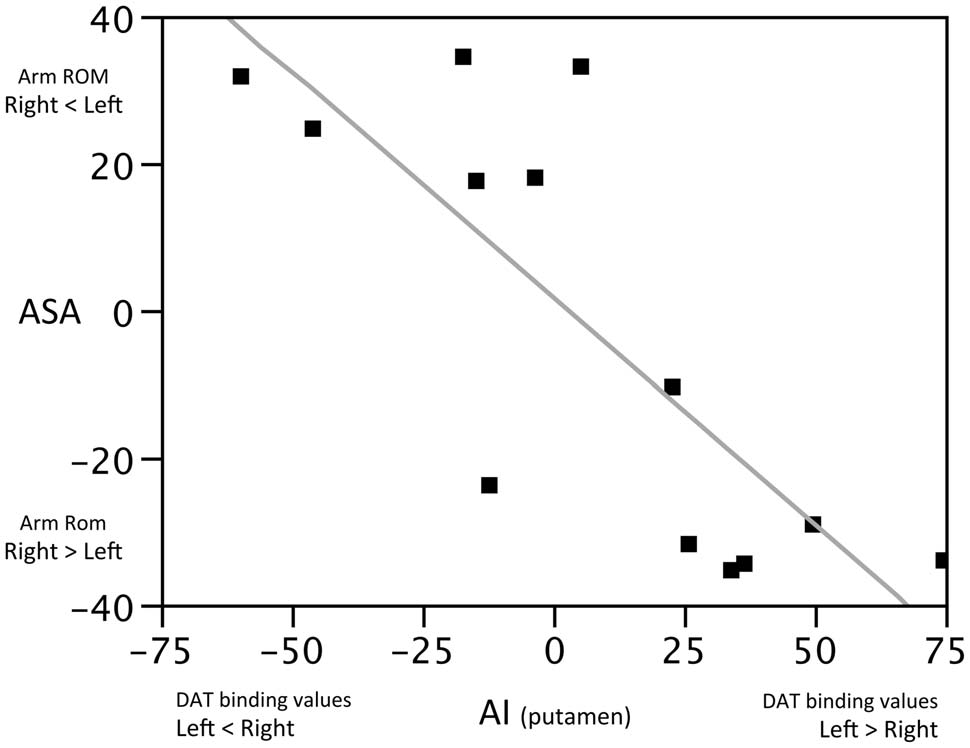
ASA significantly correlated to the AI of DAT binding values of the putamen (see, Methods).

Average ASA value was 29 (range: 18–35) for PD and 6 for HC (range: 4–12). ASA and AI indices of both the caudate nucleus (p = 0.005, RSquare = 0.52) and the putamen (Figure 1; p = 0.001; RSquare = 0.62) were strongly correlated. This correlation proved to be statistically significant also when weighted for UPDRSrb and UPDRS-III scores. Last, no correlation was found between UPDRSrb and UPDRS-III scores and arm ROM.

### Walking at Different Speeds

When walking at different velocities, the range of speeds was comparable across subjects and PD patients and large enough to reliably compute a slope line.

No difference was found for lower limbs spatio-temporal gait parameters (i.e. stride length, stride time and stance) between patients and controls also when walking at different gait velocities.

**Figure 2:**
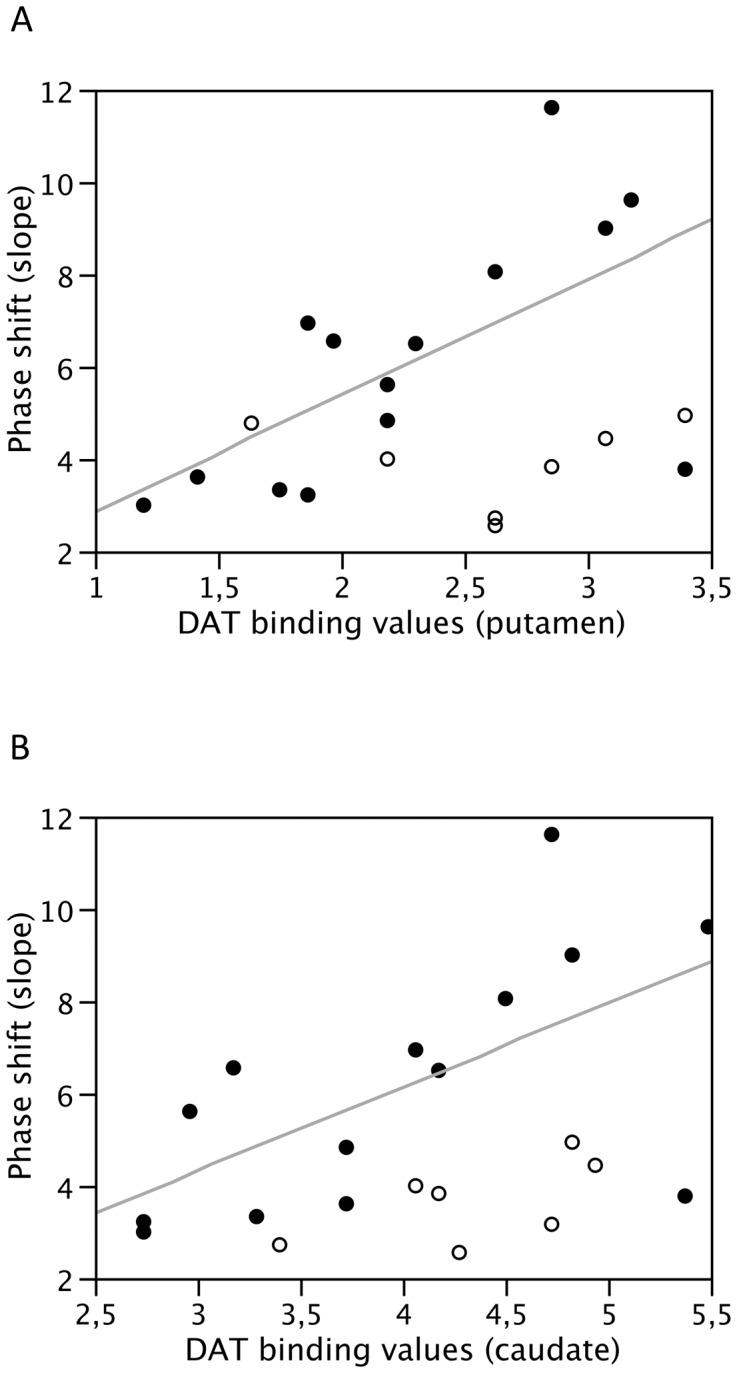
Correlation between phase shift modulation at different gait velocities and DAT binding values of the putamen (A) and caudate nucleus (B). Full dots represent arms with reduced (<18°) ROM when walking at preferred gait speed. Empty dots shows arms with arm ROM within the normal range. When arm ROM was reduced, a positive correlation was found between upper-lower limb phase shift modulation and both DAT binding values of putamen (RSquare = 0.37, p = 0.01) and caudate nucleus (RSquare = 0.38, p = 0.01). Correlation lines for arms with normal ROM (>18°) are not shown.

Phase shift modulation (slope) was significantly higher in PD patients than controls (p<0.05, Table 1). All patients were able to normally modulate all other spatio-temporal gait parameters and arm ROM (both ipsilateral and contralateral) when walking at different velocities (Table 1). Interestingly, patients with reduced arm ROM (<18°) when walking at preferred gait speed showed a significantly higher capability of modulating upper-lower limb phase shift which positively correlated with the corresponding dopaminergic content of the putamen (RSquare = 0.37, p = 0.01) and caudate nucleus (RSquare = 0.38, p = 0.01). This correlation was not present if arm ROM was in the range of normality when walking at preferred gait speed (Figure 2A and B).

## Discussion

Some relevant conclusion can be drawn from the present study: (1.) We confirmed that early-stage PD patients may exhibit normal spatio-temporal gait parameters [16]. The presence of normal lower limb locomotor automatisms in subjects with reduced arm ROM supports the notion that both types of movement may be differentially organized [17].

(2.) We did not find a linear correlation between arm ROM reduction and corresponding putaminal dopaminergic depletion. Rather, we were able to define a cut-off value for dopaminergic putaminal innervation loss before arm ROM would decrease. (3.) Inter-limb synergies might be influenced by the imbalance of dopaminergic striatal tone between the two hemispheres as shown by the strong correlation between ASA and AI indexes. These findings question a prominent unihemispheric control of arm swing during walking. Still, the ASA index should be carefully interpreted as possibly related to the cut-off itself. Indeed, in all but two patients with arm ROM unilaterally reduced, the arm with reduced ROM (according to our reference value of 18°) was contralateral to the putamen with greater dopaminergic innervation loss. (4.) When walking at different gait velocities, arms with reduced ROM showed an upper-lower limb coupling (phase shift) influenced by putaminal dopaminergic innervation. (5.) Locomotor synergies were independent of the lateralization of akinetic-rigid symptoms. Such a discrepancy may provide preliminary evidence for a different central organization of these entities in PD patients. Indeed, bradykinesia and rigidity are mainly related to the thalamo-cortical-basal ganglia loop with strict lateralized organization. [21], [22] Conversely, limbs coordination, especially during automatically performed motor task, may be related to inter-hemispheric projections of basal ganglia and possibly involve also mesencephalic centers such as the pedunculopontine nucleus (PPN) and the reticular system [23].

Limitations in our study must be considered. We arbitrarily excluded patients with abnormal spatio-temporal parameters at lower limbs to possibly avoid upper limbs related changes. By doing this, we limited the patient sample and neglected well known PD-related gait disturbances, including abnormal timing of gait and stride-to-stride variability [24]. Another limit of this study is that PD patients were not drug-naïve. Still, the 3-day wash-out as well as the several inclusion criteria support the assumption that the enrolled PD patients well represent an *in vivo* model of dopaminergic deficit and allowed us to selectively investigate the role of intrinsic dopamine and the striatum in upper limbs locomotor synergies. Last, we cannot exclude in this study a direct role of a dopaminergic spinal innervation originating from the dorsal posterior hypothalamus (A11 region) on locomotor-related movements. Beside local hypothalamic connections, projections to the neocortex and to the serotonergic dorsal raphe, A11 neurons descend as the sole source of spinal dopamine mainly through the dorsolateral funiculus [25] and innervate most heavily the superficial sensory-related dorsal horn and the intermediolateral nucleus [26]. A loss of A11 neurons might eventually alter a possible interplay between dopamine and serotonin at a spinal level and result in loss of modulation during locomotion-like activity [27]. The role of the A11 neurons in the pathophysiology of PD and in locomotion in general, has not been explicitly tested.

From a anatomo-physiological perspective, the gait-related pendular motion of upper extremities is a subconsciously and automatically performed motor task. Inter-limb coordination remains stable despite changes of limb segment mass, suggesting independence from peripheral mechanism [28] and it is maintained across kinematically and kinetically different tasks, thus possibly related to a common neural control [29].

Descending pathways responsible for the control of locomotor limb movements, can be ascribed to direct cortical-motoneuronal input and indirect pathways of the basal ganglia [30], [31], [32]. Preliminary evidence suggest that dopaminergic neurons play an important role in the execution of self-determined movements [33], in the automatic nature of the rhythmic bilateral movements of the lower-limbs [34] and the persistence of gait execution [9]. This study provides additional information to disentangle a putative role of dopamine and the striatum in locomotor synergies. We suggest an interhemispheric rather than unihemispheric influence on inter-limb coupling. This may be particularly evident when dopaminergic striatal innervation is greatly reduced (>47% dopaminergic putaminal innervation loss). Furthermore, when arm ROM is reduced, the modulation of upper-lower limb coupling (phase shift) is also related to dopaminergic striatal content.
